# The gap in life expectancy and lifespan inequality between Iran and neighbour countries: the contributions of avoidable causes of death

**DOI:** 10.1186/s12939-022-01683-8

**Published:** 2022-06-08

**Authors:** Kasim Allel, Mohammad Hajizadeh, Ali Kiadaliri

**Affiliations:** 1 Institute for Global Health, University College London, 30 Guilford Street, London, WC1N 1EH UK; 2 School of Health Administration, Dalhousie University, Halifax, Canada; 3 Clinical Epidemiology Unit, Department of Clinical Sciences Lund, Orthopaedics, Skåne University Hospital, Lund University, Remissgatan 4, SE-221 85 Lund, Sweden; 4 Centre for Economic Demography, Lund University, Lund, Sweden

**Keywords:** Life expectancy, Lifespan inequality, Avoidable causes of death, Iran, Turkey, Qatar, Kuwait

## Abstract

**Background:**

Healthcare system and intersectoral public health policies play a crucial role in improving population health and reducing health inequalities. This study aimed to quantify their impact, operationalized as avoidable deaths, on the gap in life expectancy (LE) and lifespan inequality (LI) between Iran and three neighbour countries viz., Turkey, Qatar, and Kuwait in 2015–2016.

**Methods:**

Annual data on population and causes of deaths by age and sex for Iran and three neighbour countries were obtained from the World Health Organization mortality database for the period 2015–2016. A recently developed list by the OECD/Eurostat was used to identify avoidable causes of death (with an upper age limit of 75). The cross-country gaps in LE and LI (measured by standard deviation) were decomposed by age and cause of death using a continuous-change model.

**Results:**

Iranian males and females had the second lowest and lowest LE, respectively, compared with their counterparts in the neighbour countries. On the other hand, the highest LIs in both sexes (by 2.3 to 4.5 years in males and 1.1 to 3.3 years in females) were observed in Iran. Avoidable causes contributed substantially to the LE and LI gap in both sexes with injuries and maternal/infant mortality represented the greatest contributions to the disadvantages in Iranian males and females, respectively.

**Conclusions:**

Higher mortality rates in young Iranians led to a double burden of inequality –shorter LE and greater uncertainty at timing of death. Strengthening intersectoral public health policies and healthcare quality targeted at averting premature deaths, especially from injuries among younger people, can mitigate this double burden.

**Supplementary Information:**

The online version contains supplementary material available at 10.1186/s12939-022-01683-8.

## Introduction

There is an observed increase in life expectancy (LE) in low-and-middle-income countries between 2000 and 2015, specifically in Africa from 52.7 to 62.5 years and Eastern Mediterranean Region (EMR) from 65 to 68.4 years [1]. Iran, specifically, has reported a dramatic reduction in mortality rates over the last 40 years, exhibiting one of the highest LE in the Middle East, reaching 81.6 and 76.1 years in females and males in 2019, respectively [2]. However, these improvements in LE are not shared equally by all individuals in a society. In other words, LE reflects average mortality level (i.e., higher LE implies lower mortality levels) and conceals significant variations in length of life. In fact, populations with the same LE might have marked differences in ages at death [3]. Therefore, it is important to complement LE by the lifespan inequality (LI), which accounts for the age heterogeneity at death by capturing the population differences in lifespan [4, 5].

While reducing mortality at any age would increase LE, improvements below (above) a threshold age of death will decrease (increase) LI [6]. In other words, LI decreases if more lives are saved at earlier rather than older ages. Previous studies have investigated these concepts primarily in high-income countries [4, 6–9]. These studies have shown a direct correlation between LE and LI, which is as strong as the progress in saving lives at younger ages [3]. Although, some exemptions have been observed, for instance when the population is divided into socioeconomic groups [3]. Even though mortality rates change differently among age groups (widespread variation between new-borns, middle ages, and elderly), the most successful countries in adverting premature deaths have consistently larger LE [9].

Broader outreach in health services and living standards (sanitation, hygienic practices, detection of tuberculosis, etc.) have amplified LE in the Middle East between 1995 and 2010 [10]. However, these improvements in LE are not evenly distributed across countries in the region [10]. Furthermore, despite improvements in LE, avoidable causes of death – premature deaths that could be avoided through effective public health policies and quality healthcare– still account for a large portion of all deaths in the region [11]. Indeed, a recent study identified the Middle Eastern countries among the worst performing countries in terms of premature avoidable mortality from non-communicable diseases between 1990 and 2017 [11]. The concept of “avoidable mortality” is used as a potential indicator of the influences of public health policies and healthcare quality on population health and to identify potential areas for improvement [12, 13]. Recent evidence suggests that avoidable causes of death contribute substantially to gains in LE over time as well as inequality in LE among different sociodemographic groups [14–16]. However, there exists limited evidence on their contributions to LE gain and inequality across Middle Eastern countries, where divergent access and public health policies have been in place. Moreover, there are significant variations across these countries’ population and causes of death profile as well as the quality of and access to healthcare [10]. To fill this knowledge gap, this study aimed to indirectly quantify the contributions of quality healthcare and public health policies, operationalised as avoidable deaths, on the cross-country gap in LE and LI between Iran and three neighbour countries viz., Kuwait, Qatar, and Turkey for the 2015–2016 period. Our results can be used to help tailoring healthcare and intersectoral public policies in Iran for a more equitable improvement in LE in Iran.

## Methods

### Data source

Our study’s sample is comprised of Iran and three neighbour countries (Kuwait, Qatar, and Turkey) with data available on causes of death in the World Health Organization (WHO) mortality database for the years 2015–2016 (http://www.who.int/healthinfo/mortality_data/). We extracted the annual data on underlying causes of death by age (0, 1–4, 5–9, …,85+) and sex from the WHO mortality database. The WHO mortality database includes all medically certified deaths as reported periodically on an annual basis by the member countries based on each country’s civil vital registration system (CVRS). Causes of death are reported with the official International Classification of Diseases (ICD) codes. The WHO verifies the quality of these data to ensure comparability and reliability, but no adjustments for underreporting are made. We aggregated the data for the whole 2-year period to avoid random year by year fluctuations. We then classified the causes of death as avoidable death using the list developed by the statistical office of the European Union (Eurostat) and the Organisation for Economic Co-operation and Development (OECD) [17]. We further split the causes of death into five mutually exclusive groups: only treatable (e.g., childbirth), only preventable (e.g., injuries, drug- and alcohol-related deaths), both treatable and preventable (e.g., diabetes mellitus), ischaemic heart disease (IHD), and non-avoidable deaths. The former four groups represent avoidable causes of death. Treatable causes of death include deaths that “in the light of medical knowledge and technology at the time of death, all or most deaths from that cause could be avoided through good quality healthcare”, while preventable deaths are those deaths that “in the light of understanding of the determinants of health at the time of death, all or most deaths from that cause (subject to age limits if appropriate) could be avoided by public health interventions in the broadest sense” [17]. A full definition of each category, their respective ICD-10 codes and corresponding subgroup causes can be found elsewhere [17]. In this paper, we refer to public health policies as intersectoral strategies covering healthcare, education, economics, road safety, welfare, among others. Avoidable causes were capped at 74 years of age and deaths among older individuals were considered as non-avoidable.

### Statistical analyses

We estimated sex-specific LE at birth for each country using abridged life tables for the study period [18]. We employed a continuous-change model [19] to quantify age- and cause-specific contributions into the gap in LE and LI between Iran and the other countries. We considered Iran as the reference country; hence, contributions into greater (smaller) LE and LI in Iran will be positive (negative). We used the standard deviation (SD) of the distribution of age at death as a measure of LI [8, 20]. The SD is an absolute measure of LI meaning that the LI would be unaffected by equal absolute change in everyone’s lifespan [21]. All data preparation and visualization were implemented in Stata version 17 and data analyses were conducted using the R software utilising publicly available codes from the following source: https://github.com/jmaburto.

### Sensitivity analyses

Given the variation in countries’ CVRS coverage, we conducted a series of sensitivity analyses to assess the impact of the data source used on our estimates. We re-estimated our models using age-specific mortality rates from the life tables of following sources for the year 2015: The Institute for Health Metrics and Evaluation (IHME) which is responsible for the Global Burden of Disease (GBD) Study [22], United Nations (UN) [23], and WHO [1]. This means that age- and cause-specific mortality rates were adjusted to reflect age-specific all-cause mortality rates reported in life tables from IHME, UN, and WHO. This was done through dividing the all cause age-specific number of deaths in WHO mortality database on age-specific mortality rates from these life tables to estimate the population sizes for each age group (this is the population size that would yield the same age-specific mortality rates as the life table of interest). We then modified the cause-specific mortality rates in each age group based on these new population estimates. For example, if all-cause and cause A number of deaths were 30,369 and 2853 in age group 70–74 in WHO mortality database and the mortality rate for this age group was 0.0298 in IHME life table, we divided 30,369 on 0.0298 to obtain a population estimate of 1,019,094. We then divide 2853 on 1,019,094 to obtain an age-specific mortality rate for cause A that reflects the IHME life table.

## Results

### Overview of LE & LI

Overall, LEs for Iranian females and males were 80.0 and 76.2 years, respectively, during 2015–2016. In both sexes, the greatest and narrowest differences were seen compared with people in Qatar and Turkey, respectively (Table 1, panel A). On the other hand, LI was greatest in Iran compared to the other countries ranging from 1.1 years gap with Turkey among females to 4.5 years gap with Qatar among males (Table 1, panel B).

**Table 1:** Summary of life expectancy and lifespan inequality by country and sex

Country/sex	Males	Difference ^a^	Females	Difference ^a^
**A. Life expectancy (LE)**				
Iran	76.2	–	80.0	–
Kuwait	80.2	−4.0	81.0	−1.1
Qatar	82.8	−6.6	85.2	−5.3
Turkey	75.6	+ 0.5	81.0	−1.0
**B. Lifespan inequality (LI)**				
Iran	18.5	–	16.1	–
Kuwait	15.1	+ 3.4	12.8	+ 3.3
Qatar	14.0	+ 4.5	14.6	+ 1.5
Turkey	16.2	+ 2.3	15.0	+ 1.1

^a^Differences were computed comparing Iran’s sex specific LE or LI with the other countries

#### LE and LI decomposition

##### Iran versus Kuwait

**Fig. 1:**
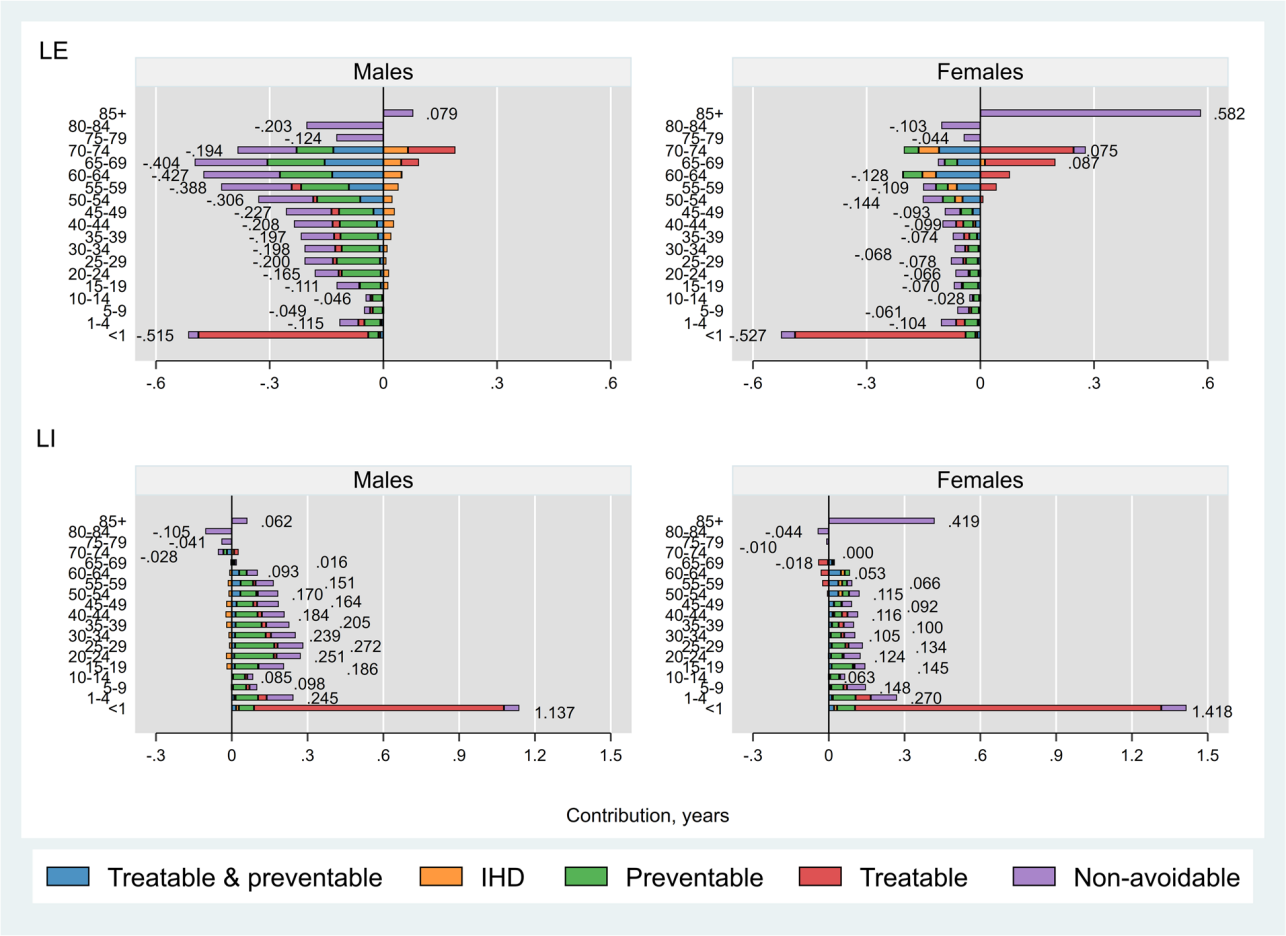
Age- and cause-specific contributions to differences in life expectancy (LE) and lifespan inequality (LI) between Iran and Kuwait, by sex. Positive (negative) values indicate age-specific contributions to larger (lower) LE and LI in Iran compared with Kuwait. Numbers indicate the total age-specific contributions. IHD: Ischaemic Heart Disease

Iranian males experienced higher age-specific mortality rates than males in Kuwait across all but the oldest age group (negative age-specific contributions to LE, Fig. 1 upper left panel). Among females, Iran performed better in age-groups 64–74 and 85+ years, specifically there were 0.6 years LE advantage from non-avoidable deaths in females ≥85 years (Fig. 1 upper right panel). Avoidable causes of death accounted for 2.2 (out of 4.0) years and 1.1 (out of 1.1) years of lower LE in Iranian males and females, respectively, compared to Kuwait. Among avoidable causes, maternal/infant mortality had substantial contributions to LE disadvantage in Iran (around 0.4 years in each sex). On the other hand, preventable causes (mainly injuries) had the greatest contributions to LE disadvantage in Iran among males aged 1–59 years (about 1.0 year) and females aged 1–49 years (about 0.3 years). Moreover, lower mortality from treatable causes among females aged 50–74 years and men aged 65–74 years and from IHD among men aged 15–74 years contributed to LE advantages in Iran versus Kuwait.

Overall, avoidable causes contributed to 2.4 (out of 3.4) years and 2.2 (out of 3.3) years higher LI in Iranian males and females, respectively, compared with their counterparts in Kuwait (Fig. 1 bottom panels). Injury-related deaths and infant mortality had the greatest contributions to the LI gap among males (0.9 years each) while maternal/infant mortality was the leading contributor to the LI gap (1.2 out of 3.3 years) among females. Higher mortality in age groups< 70 years contributed to higher LI while opposite was observed in age groups≥70 years.

##### Iran versus Qatar

**Fig. 2:**
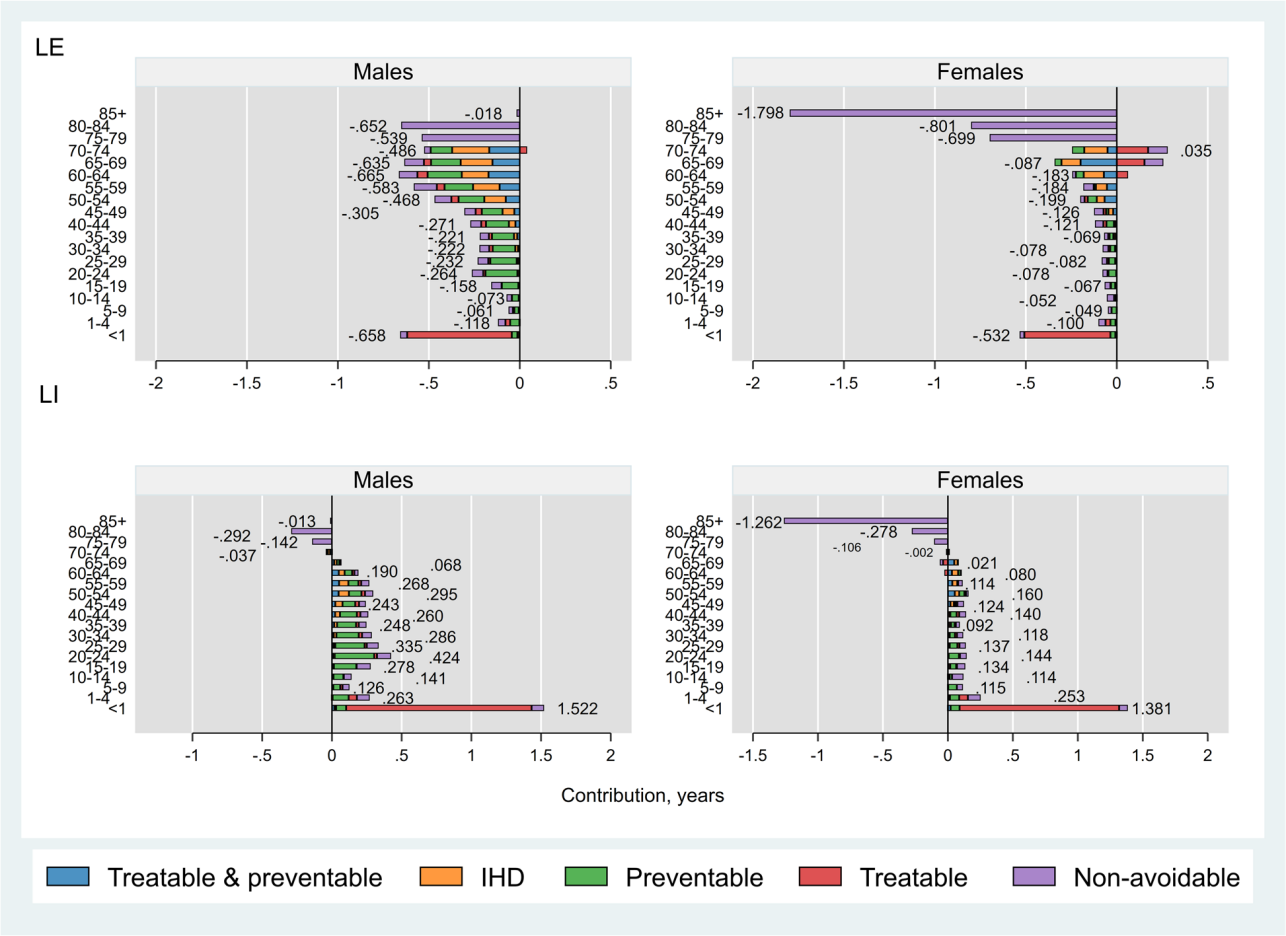
Age- and cause-specific contributions to differences in life expectancy (LE) and lifespan inequality (LI) between Iran and Qatar, by sex. Positive (negative) values indicate age-specific contributions to larger (lower) LE and LI in Iran compared with Qatar. Numbers indicate the total age-specific contributions. IHD: Ischaemic Heart Disease

Among males, almost all age- and cause-specific contributions to LE were negative, suggesting higher mortality rates in Iran compared to Qatar (only exception was deaths from treatable causes in age group 70–74 years, Fig. 2 upper left panel). While similar patterns were seen in females, lower mortality from treatable and non-avoidable causes in females aged 65–74 years resulted in LE advantage in these two age groups in Iran versus Qatar (Fig. 2 upper right panel). Avoidable causes of death accounted for 4.4 (out of 6.6) years and 1.7 (out of 5.3) years lower LE among males and females, respectively, in Iran compared with Qatar. In both sexes, treatable causes and preventable causes were the leading contributors of LE disadvantage in Iran in age groups < 1 year and 1–44 years, respectively, across avoidable causes of death. While preventable causes had a greater contribution to LE disadvantage among Iranian males than other avoidable causes, treatable & preventable causes had greater contribution among females.

Avoidable causes contributed to 4.0 (out of 4.5) years and 2.4 (out of 1.5) years of greater LI among males and females, respectively, in Iran compared to Qatar (Fig. 2 bottom panels). Overall, all causes but non-avoidable causes in females, contributed to a greater LI in Iran versus Qatar. While among males, preventable causes and treatable causes had similar contributions to greater LI in Iran (around 1.6 years each), among females’ treatable causes had the greatest contribution (1.3 years).

##### Iran versus Turkey

**Fig. 3:**
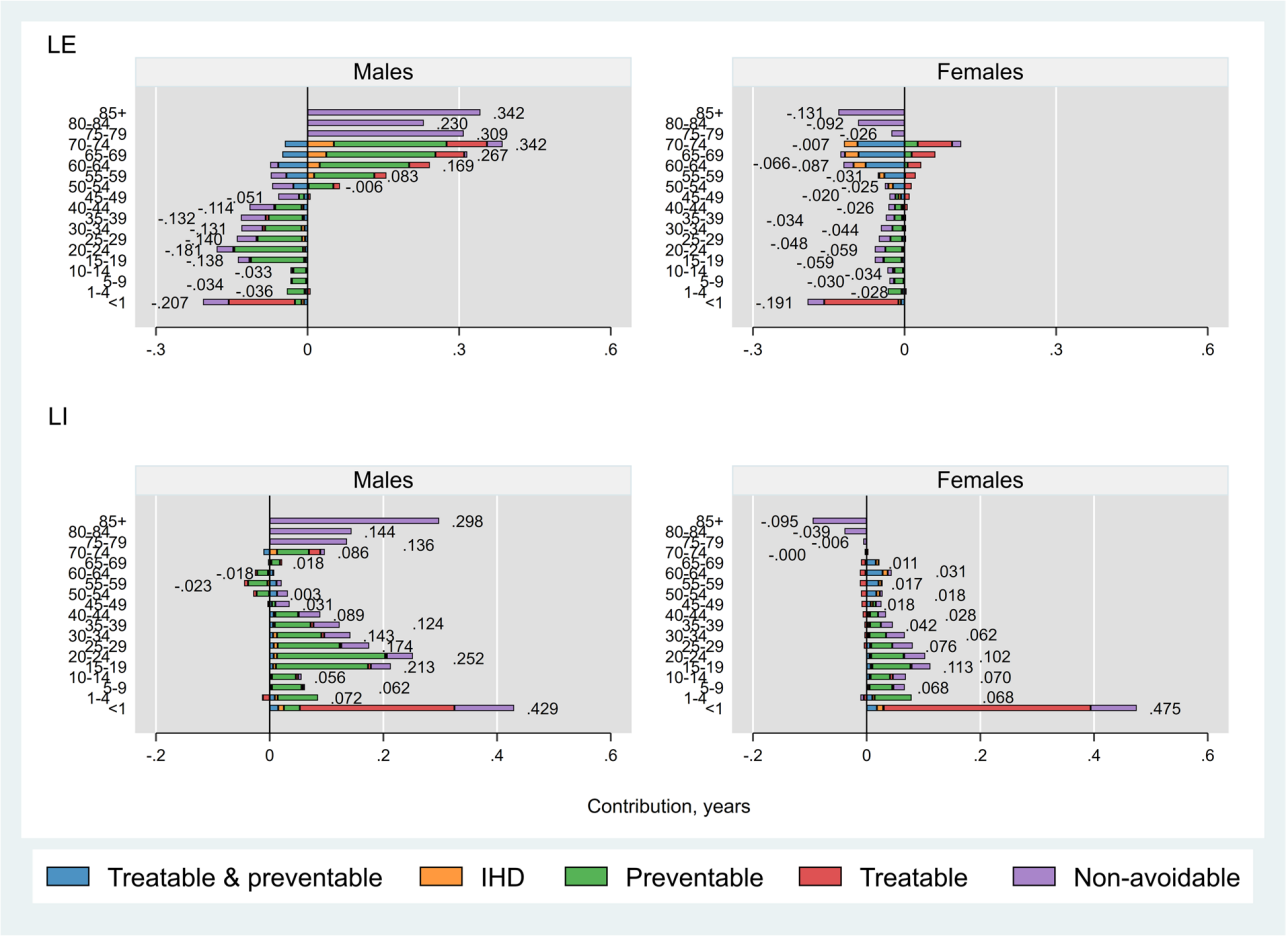
Age- and cause-specific contributions to differences in life expectancy (LE) and lifespan inequality (LI) between Iran and Turkey, by sex. Positive (negative) values indicate age-specific contributions to larger (lower) LE and LI in Iran compared with Turkey. Numbers indicate the total age-specific contributions. IHD: Ischaemic Heart Disease

While Iranian males aged < 55 years had higher mortality rates than Turkish males, the opposite was seen among older males (Fig. 3 upper left panel). In overall, non-avoidable causes of death accounted for almost all (0.5 years) LE advantage in Iranian males. Interestingly, while higher mortality rate from injuries contributed to 0.7 years lower LE among Iranian males, this was offset by 0.7 years LE advantage from lung cancer. Among females, mortality rates in all age groups were higher in Iran than Turkey (Fig. 3 upper right panel). Avoidable causes accounted for 0.6 (out of 1.0) years shorter LE in Iranian females, with injuries (0.3 years) followed by hypertensive diseases (0.2 years) and diabetes mellitus (0.2 years) as leading causes of this LE disadvantage.

Avoidable causes contributed to 1.3 (out of 2.3) years and 0.9 (out of 1.1) years higher LI among males and females, respectively, in Iran compared with Turkey (Fig. 3 bottom panels). In both sexes, preventable causes had the greatest contributions to the LI gap (0.8 and 0.4 years in males and females, respectively), driven mainly by injuries among people aged 15–35 years. Of note, since mortality from lung cancer occurred mostly among older males, it decreased LI in Iran by 0.1 years while injuries occurred mostly among younger people and increased LI in Iran by 0.8 years. Full detailed results on age- and cause-specific contributions to LE and LI are presented in the supplementary excel file Tables A1-A4.

##### Sensitivity analysis

**Fig. 4:**
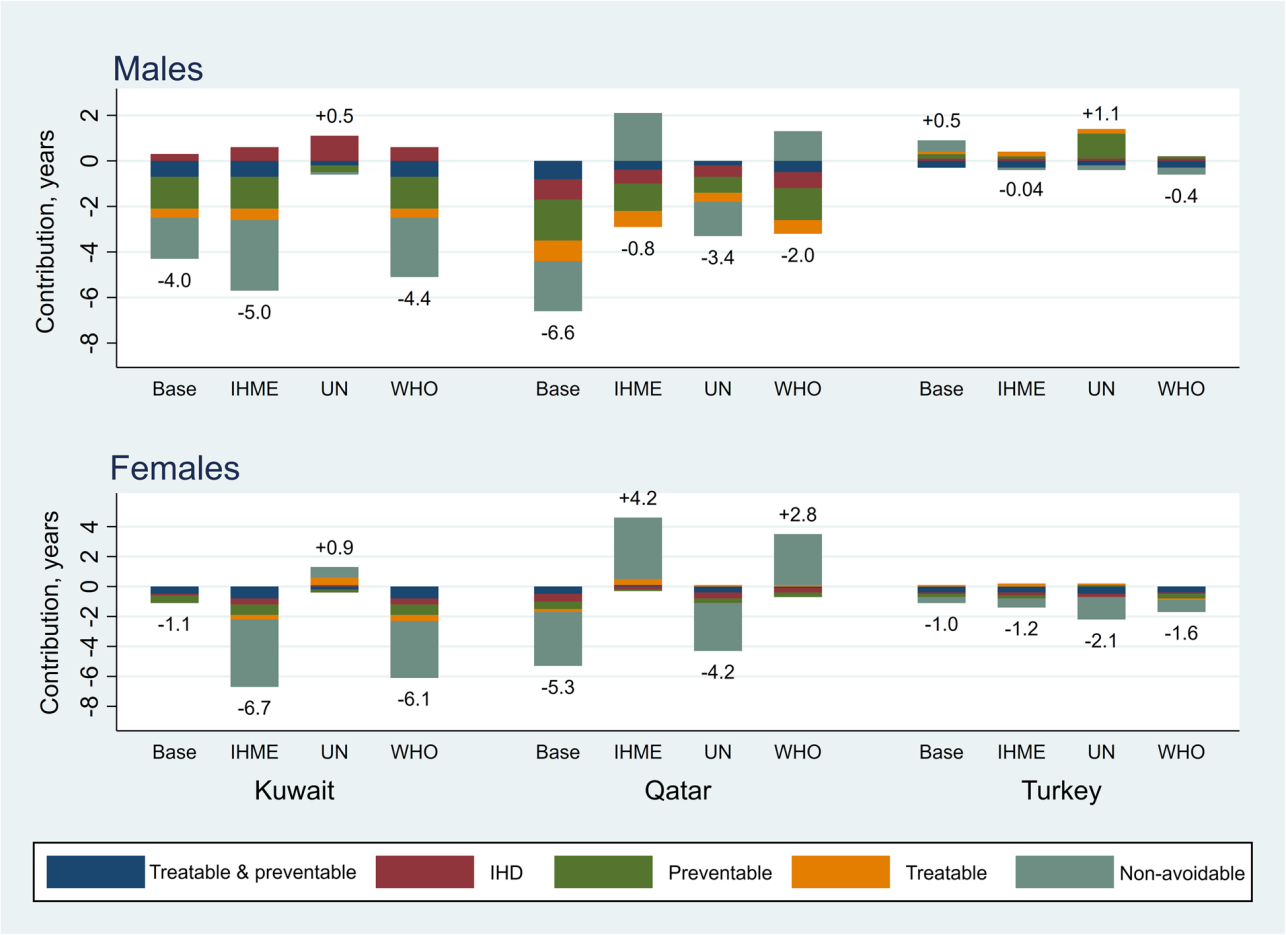
The effects of data sources on cause-specific contributions to differences in life expectancy (LE) between Iran and neighbour countries, by sex. Numbers indicate difference in LE between Iran and the neighbour countries. IHME: Institute for Health Metrics and Evaluation; IHD: Ischaemic Heart Disease; UN: United Nations; WHO: World Health Organization

**Fig. 5:**
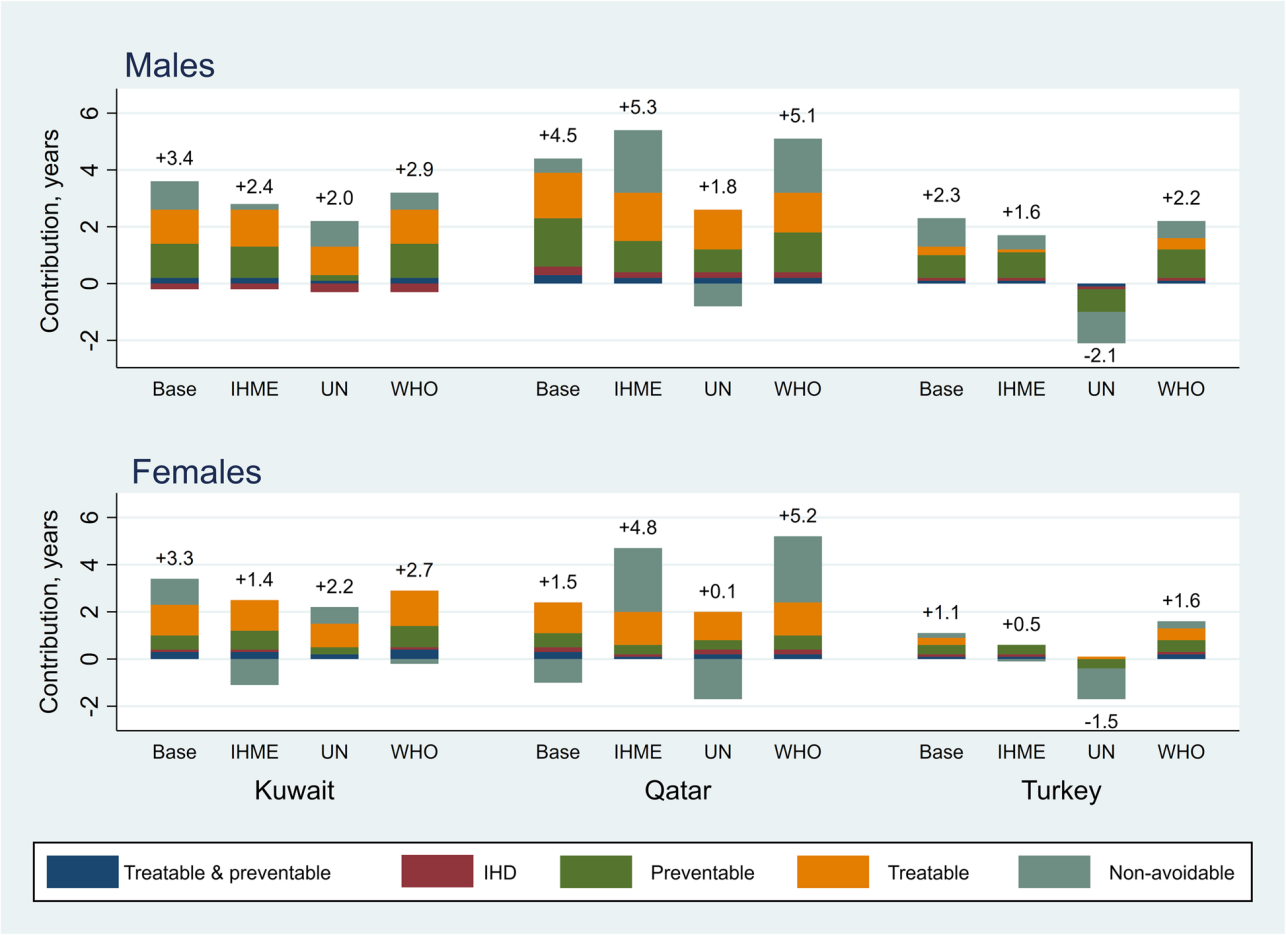
The effects of data sources on cause-specific contributions to differences in lifespan inequality (LI) between Iran and neighbour countries, by sex. Numbers indicate difference in LI between Iran and the neighbour countries. IHME: Institute for Health Metrics and Evaluation; IHD: Ischaemic Heart Disease; UN: United Nations; WHO: World Health Organization

The results of the sensitivity analysis showed that our estimates were to some extent sensitive to the data source used, even though the overall conclusions were less influenced (Figs. 4 & 5, detailed estimates are presented in supplementary excel file Tables A5-A10). In overall, the results for LE were more sensitive to the data source than those for LI. For males’ LE, the comparison with Turkey was most sensitive in which, opposed to the base analysis, in two sensitivity analyses Iranian males had smaller LE. For females’ LE, the results for Qatar were most sensitive where in two sensitivity analyses Iranian females had greater LE than females in Qatar. For LI, similar to the base analysis the sensitivity analyses also showed the greatest LI for Iranian males and females and the only exception was Iran-Turkey comparison using the UN life table.

## Discussion

The results of this comparative study revealed that avoidable causes of death had substantial contributions to Iran’s lower LE and higher LI compared to the three neighbour countries in the region during 2015–2016. We also observed variations in cause-specific contributions by age and sex. For instance, while preventable causes of death had generally higher contributions than other avoidable causes of death among males, their contributions were comparable with other causes in females. In addition, Iran generally performed better on treatable causes of death among people aged 60+ years. Higher maternal/infant mortality and injuries-related deaths resulted in higher uncertainty in timing of death among Iranians than their counterparts in the three neighbour countries.

Substantial contributions of avoidable causes of death to the cross-country gap in LE observed in this study is in line with few previous studies conducted in other parts of the world [15, 24]. Higher mortality rates from avoidable causes in Iran compared with the three neighbour countries studied here is consistent with the findings from the GBD study on Healthcare Access and Quality Index, an index based on 32 treatable causes of death, where Iran had the lowest score (71.8 out of 100) compared with Qatar (81.7), Kuwait (80.7) and Turkey (74.4) in 2016 [25]. While a previous study conducted in the UK reported that preventable causes had the greatest contributions to the cross-country gap in LE compared with other avoidable causes in both sexes [15], this was only the case among males in the current study. This might partially be attributable to cultural differences between the UK and the Middle Eastern countries in females’ roles in the society. For example, drug- and alcohol-related deaths contributed to 11.2 to 14.7% of the LE gap in females across the countries in the UK [15], while their contributions were 0.7 to 1.8% in this study. Moreover, the contributions of maternal/infant mortality were negligible in the UK [15], whereas they were among leading causes of LE gap among females in this study.

Within avoidable causes, injuries were the leading cause of LE disadvantage in all pairwise comparisons among Iranian males, and compared to Turkey among Iranian females. Indeed, injuries alongside diabetes mellitus, hypertensive diseases, other treatable and preventable causes, drug-related death and maternal/infant mortality were the only causes that contributed to LE disadvantage in Iran in both sexes in all pairwise comparisons. Importantly, since injuries-related deaths are more common among younger age groups, they were a leading cause of LI disadvantage among Iranian males and females. These findings are not surprising given the fact that the age-standardized mortality rate caused by road traffic injury in Iran is one of the highest worldwide (22 deaths per 100,000 population in 2019), surpassing Kuwait, Turkey, and Qatar (15, 7, and 7 deaths per 100,000 population, respectively) [26, 27]. In particular, males are disproportionately affected, with road traffic injuries being the second leading cause of disability-adjusted life years (DALYs) among Iranian males compared with 6th, 8th and 10th leading cause of DALYs in Qatar, Kuwait and Turkey, respectively, in 2015 [28]. The higher burden of road traffic injuries in Iran compared with the neighbouring countries might be due to poor road safety, poor quality and safety of cars, inadequate public transportation, risky driving behaviours and low adherence to driving regulations (e.g. the use of seatbelt and helmet), as well as inadequate access to high quality trauma care system [29]. Given the substantial contributions of injuries to LE and LI disadvantages in Iran, urgent intersectoral interventions (beyond the healthcare system) such as re-organisation of traffic laws, transportation infrastructure, and controlling the manufacturing industry are strongly needed [30–32]. The WHO non-communicable diseases national plan, together with Iran’s Road Safety Strategy Plan, has set a target of 20% relative reduction in deaths due to traffic injuries by 2025 in the country [33] and several interventions including more stringent regulations (e.g. compulsory seat belt and speed limit laws), increased fines for traffic violations, random drug and alcohol testing, and improvements in the roads network construction have been implemented [29]. Although these actions resulted in a decline in road traffic injuries in recent years in Iran [34], these injuries still incur a high burden and Iran is unlikely to achieve the targeted reduction in the national plan. In comparison, Qatar has introduced a trauma system that provides emergency care to every citizen and non-citizen, including education, diagnosis, treatment, rehabilitation, and community reintegration of the injured which resulted in substantial reductions in deaths attributable to road traffic and injuries [35]. Qatar has also invested significantly in upgrading the roads and railways-related services together with reducing the pedestrian accidents through the Decade of Action for Global Road Safety [36]. Turkey is another good example given the solid enforcement of the laws against blood alcohol concentration and driving national standards including enforced regulations on passengers’ protection [37]. Turkey launched a six-pillar program called the “New Approaches, Targets and Solutions on Road Traffic Safety” through an intersectoral strategy involving population education, enforcement, support to traffic services, information, motivation of personnel, and legislative matters [38]. It resulted in a 20% decrease in road injuries in 2010 after 3 years of its implementation [37, 38]. All these measures promoted by Qatar and Turkey can be used as a benchmark for Iran.

Besides injuries, maternal/infant mortality was another leading cause of LE and LI disadvantages in Iran. There were 14.7 deaths per 1000 living births (95% confidence interval [CI]: 10.8–19.5) in Iran in 2015 [39], right below the average of the EMR (44.2, 95%CI: 41.6–46.9), but almost doubling the rate of the six countries having achieved Millennium Development Goal 4 by 2015 in the region (e.g., 8.2[95%CI: 6.5–10.2] and 8.6 [95%CI: 6.0–12.1] in Kuwait and Qatar, respectively) [40]. Higher rates of maternal/infant mortality offset lower mortality rates for other treatable causes, especially infectious diseases and diseases of the respiratory system in Iran, resulting in an overall negative contribution from treatable causes into LE in Iran in three pairwise comparisons. This higher maternal/infant mortality rates might be due to lower access to high quality maternity care, delayed emergency care provision, high rates of caesarean section deliveries, and lower socioeconomic status especially financial hardship experienced in the recent decade following sanctions on Iran [41–44]. Specifically, for caesarean delivery, Iran has the second highest caesarean rate in the EMR (48%, right below Egypt with 52%), while Kuwait and Qatar have 12 and 20%, respectively. The ease of access to facility-based delivery, women’s fear of labour pain, and clinicians’ convenience, financial gain and fear of litigation have been explained as the main drivers [45, 46]. Although Iran has experienced steep reductions in maternal/infant mortality in recent decades, it still suffers from higher mortality rates than the neighbouring countries. This highlights the need for further actions including promoting maternal education and improving access to antenatal and postnatal care [42–44]. In particular, there is a recent shift in family planning policies toward rising restrictions on access to abortion, contraception and birth limiting surgeries in Iran [47], which might lead to increased maternal/infant mortality and in turn widening the gap in LE and LI with other countries in the region.

Our results suggested that while IHD was generally associated with LE advantage for Iranian males, it contributed to LE disadvantage for Iranian females compared with their counterparts in the neighbouring countries. Iran is among the countries with the highest rates for cardiovascular diseases (CVD) exhibiting more than 9000 age-standardised cases of CVD per 100,000 persons [48]. IHD has also the highest disease burden, accounting for 26% of total deaths in the country, and with higher incidence rates in females than in males among people aged 70+ years [49]. In addition, a recent study from Isfahan province in Iran reported a greater rise in IHD incidence among females than males during recent two decades [50]. Although it should be noted that despite LE disadvantage from IHD for Iranian females in our cross-country comparison, IHD mortality rates are higher for males than females in Iran. Our results possibly reflect greater cross-country differences in IHD’s risk factors (e.g. hypertension, metabolic syndrome, obesity, socioeconomic and cultural distress, unhealthy lifestyle, low affordability, poor accessibility to primary healthcare) [50] for females compared with males. For instance, while the age-standardised prevalence of hypertension (19.6%) and dyslipidaemia (58.1%) is higher among Iranian females compared to females in Kuwait (15 and 55.7%) and Qatar (13.6 and 57.6%), the prevalence is generally lower in Iranian males (21.2 and 41.8%) than males in Kuwait (23.1 and 56.2%) and Qatar (19.6 and 56.8%) [51]. Moreover, we speculate that population-based interventions and intersectoral public health policies implemented in Iran in the recent decades might have benefited Iranian males more substantially than Iranian females either due to higher rates of IHD mortality among males or due to unequal access to these interventions. Further analyses are needed to explore the underlying causes of higher IHD mortality rates among Iranian females compared with females in the neighbouring countries.

Avoidable causes could also be prevented if healthcare systems are strengthened. Iran has improved towards universal health coverage (UHC) through the coordination of the Ministry of Health and Medical Education, but free health insurance coverage is not yet a reality for secondary and tertiary health services [52]. In contrast, Qatar, Kuwait and Turkey have national health insurance schemes with a state-fund healthcare system providing free access and treatment to the primary services, avoiding excessive out-of-pocket expenditures (OOP) [53–56]. The significant share of OOP (35%) from total healthcare expenditure [56], derived from the dual organisation of the healthcare system (public and private) with the private part providing vast healthcare services, also contributed to socioeconomic inequalities in healthcare use and mortality rates in Iran.

Our study is subject to some limitations. First, the OECD classification utilises an age threshold of around 75 years to calculate avoidable (premature) deaths, which might not mirror specific countries’ characteristics. Deaths in people above 75 years old are not considered avoidable, even though these could have been avoided through the correct prevention or treatment. Future analyses should incorporate specific age-targeted definitions of avoidable causes of death. Second, the causes of death are treated as mutually exclusive although they may be linked to each other. Third, death registration and certifications systems might confront different completeness rates and coding practices, which could bias mortality outcomes and our study findings. For instance, WHO reports that completion rates for death counts provided by countries’ CVRS were 90, 50, 50, 91% for Iran, Kuwait, Qatar and Turkey, respectively [57]; similar to those reported by the GBD [58]. These discrepancies in countries’ CVRS quality and completeness might be partially responsible for the estimated contributions of avoidable causes of death to cross-country differences in LE/LI in our study. While it is hard to quantify the magnitude of the bias, these problems call for great caution in interpreting our findings. Also, we acknowledge that our analyses were sensitive to the data source used, exhibiting variations in the age structure of all-cause mortality and consecutively on age-specific contribution to LE/LI. The estimates from IHME and UN are modelled estimates relying on assumptions that might not accurately capture the distributions of deaths by age, sex, and cause [59, 60]. For example, the IHME’s estimates rely on the availability of high quality mortality data and when such data for a location, time, age group or cause is not available, the model borrows the data from other sources which include mainly data from high-income countries [59, 61]. However, such data might not accurately represent the distribution of deaths in other locations particularly low- and middle-income countries. In addition, to our knowledge, no comparable data on causes of death by ICD-10 codes are publicly available in other sources, including the IHME and UN. However, raw data from CRVS used in WHO mortality database are prone to coding errors, misclassification bias, imprecise causes of death (“ill-defined” death), and incomplete coverage which can bias the age- and cause-specific contributions to LE/LI [61]. Therefore, complementing WHO mortality data with age-specific deaths estimated from other sources such as IHME, as has been done in the present study, would provide better insights on age- and cause-specific contributions to cross-country gaps in LE/LD. Fourth, avoidable mortality may be indirectly related to the contribution of intersectoral public health policies in each country from a non-causal perspective, but not directly. Finally, even though the countries selected are neighbours, they may differ in their characteristics (e.g., culture and demographic composition), which represent further challenges to quantify the differences between them realistically.

## Conclusions

Our findings showed that avoidable causes of death contributed substantially to the double burden of inequality (i.e., shorter LE and higher LI) in Iran compared with three neighbouring countries. Higher rates of injury-related deaths and maternal/infant mortality among younger people in Iran are the leading causes of this double burden and urgent actions including intersectoral public health policies are needed in the country. The recent establishment of the Iranian non-communicable diseases (NCDs) committee and development of a national action plan 2015 to tackle NCDs can play a key role in achieving this [62]. In addition, a high priority should be given to promotion of maternity care including counselling and education programs to improve lifestyle and mothers’ health. Moreover, active involvement of local authorities for the success of any action towards reducing premature deaths are vital.

## Supplementary Information

**Additional file 1: Table A1.** Age- and cause-specific contributions to gap in life expectancy between Iran and the neighbour countries in males. **Table A2.** Age- and cause-specific contributions to gap in life expectancy between Iran and the neighbour countries in females. **Table A3.** Age- and cause-specific contributions to gap in lifespan inequality between Iran and the neighbour countries in males. **Table A4.** Age- and cause-specific contributions to gap in lifespan inequality between Iran and the neighbour countries in females. **Table A5.** Age- and cause-specific contributions to gap in life expectancy and lifespan inequality between Iran and the neighbour countries in males based on the life table from the institute for health metrics and evaluation. **Table A6.** Age- and cause-specific contributions to gap in life expectancy and lifespan inequality between Iran and the neighbour countries in females based on the life table from the institute for health metrics and evaluation. **Table A7.** Age- and cause-specific contributions to gap in life expectancy and lifespan inequality between Iran and the neighbour countries in males based on the life table from the United Nations. **Table A8.** Age- and cause-specific contributions to gap in life expectancy and lifespan inequality between Iran and the neighbour countries in females based on the life table from the United Nations. **Table A9.** Age- and cause-specific contributions to gap in life expectancy and lifespan inequality between Iran and the neighbour countries in males based on the life table from the world health organization. **Table A10.** Age- and cause-specific contributions to gap in life expectancy and lifespan inequality between Iran and the neighbour countries in females based on the life table from the world health organization.

## Data Availability

Data used in the study is publicly available at the IHME, UN, and WHO websites.

## Abbreviations

CVD: Cardiovascular diseases
CVRS: Civil vital registration system
EMR: Eastern Mediterranean Region
GBD: Global Burden of Diseases
ICD: International Statistical Classification of Diseases and Related Health Problems
IHD: Ischaemic heart disease
IHME: Institute for Health Metrics and Evaluation
LE: Life expectancy
LI: Lifespan inequality
NCD: Non-Communicable Diseases
OECD: Organisation for Economic Co-operation and Development
OOP: Out-of-pocket expenditures
SDGs: Sustainable Development Goals
UHC: Universal health coverage
UN: United Nations
WHO: World Health Organization

## References

[1] The Global Health Observatory https://www.who.int/data/gho/data/countries.

[2] Ebrahimi N, Mehdipour P, Mohebi F, Ghanbari A, Azmin M, Farzadfar F (2020). Improved population health in Iran from 1979 to 2019; decreasing mortality rates and increasing life expectancy. Arch Iran Med.

[3] Aburto JM, Villavicencio F, Basellini U, Kjærgaard S, Vaupel JW (2020). Dynamics of life expectancy and life span equality. Proc Natl Acad Sci.

[4] Aburto JM, van Raalte A (2018). Lifespan dispersion in times of life expectancy fluctuation: the case of central and Eastern Europe. Demography.

[5] van Raalte AA, Sasson I, Martikainen P (2018). The case for monitoring life-span inequality. Science.

[6] Aburto JM, Kristensen FF, Sharp P (2021). Black-white disparities during an epidemic: life expectancy and lifespan disparity in the US, 1980–2000. Econ Hum Biol.

[7] Zheng Y, Chen M, Yip PS (2021). A decomposition of life expectancy and life disparity: comparison between Hong Kong and Japan. Int J Health Policy Manag.

[8] Aburto JM, Wensink M, van Raalte A, Lindahl-Jacobsen R (2018). Potential gains in life expectancy by reducing inequality of lifespans in Denmark: an international comparison and cause-of-death analysis. BMC Public Health.

[9] Vaupel JW, Zhang Z, van Raalte AA (2011). Life expectancy and disparity: an international comparison of life table data. BMJ Open.

[10] Gilligan AM, Skrepnek GH (2015). Determinants of life expectancy in the eastern Mediterranean region. Health Policy Plan.

[11] Martinez R, Lloyd-Sherlock P, Soliz P, Ebrahim S, Vega E, Ordunez P, McKee M (2020). Trends in premature avertable mortality from non-communicable diseases for 195 countries and territories, 1990–2017: a population-based study. Lancet Glob Health.

[12] Nolte E, McKee CM (2008). Measuring the health of nations: updating an earlier analysis. Health Aff.

[13] Rutstein DD, Berenberg W, Chalmers TC, Child CG, Fishman AP, Perrin EB, Feldman JJ, Leaverton PE, Lane JM, Sencer DJ (1976). Measuring the quality of medical care: a clinical method. N Engl J Med.

[14] Bíró A, Hajdu T, Kertesi G, Prinz D. Life expectancy inequalities in Hungary over 25 years: the role of avoidable deaths. Popul Stud. 2021;75(3): 443–55. 10.1080/00324728.2021.1877332 33527888

[15] Allel K, Salustri F, Haghparast-Bidgoli H, Kiadaliri A (2021). The contributions of public health policies and healthcare quality to gender gap and country differences in life expectancy in the UK. Popul Health Metrics.

[16] Kiadaliri A (2021). Avoidable deaths in Sweden, 1997–2018: temporal trend and the contribution to the gender gap in life expectancy. BMC Public Health.

[17] Organization for Economic Co-operation and Development (2019). Avoidable mortality: OECD/Eurostat lists of preventable and treatable causes of death (November 2019 version).

[18] Chiang CL (1984). Life table and its applications. Life table and its applications.

[19] Horiuchi S, Wilmoth JR, Pletcher SD (2008). A decomposition method based on a model of continuous change. Demography.

[20] Wrycza TF, Missov TI, Baudisch A (2015). Quantifying the shape of aging. PLoS One.

[21] Van Raalte AA, Caswell H (2013). Perturbation analysis of indices of lifespan variability. Demography.

[22] GBD Results Tool https://ghdx.healthdata.org/gbd-results-tool?params=gbd-api-2019-permalink/65e7472150ac306db704c7f1086621fd.

[23] World Population Prospects (2019). Population Dynamics.

[24] Velkova A (1997). Wolleswinkel-Van den Bosch JH, Mackenbach JP: the east-west life expectancy gap: differences in mortality from conditions amenable to medical intervention. Int J Epidemiol.

[25] Fullman N, Yearwood J, Abay SM, Abbafati C, Abd-Allah F, Abdela J, Abdelalim A, Abebe Z, Abebo TA, Aboyans V (2018). Measuring performance on the healthcare access and quality index for 195 countries and territories and selected subnational locations: a systematic analysis from the global burden of disease study 2016. Lancet.

[26] Mortality caused by road traffic injury (per 100,000 population) https://data.worldbank.org/indicator/SH.STA.TRAF.P5.

[27] James SL, Lucchesi LR, Bisignano C, Castle CD, Dingels ZV, Fox JT, Hamilton EB, Liu Z, McCracken D, Nixon MR (2020). Morbidity and mortality from road injuries: results from the global burden of disease study 2017. Inj Prev.

[28] Sepanlou SG, Parsaeian M, Krohn K, Afshin A, Farzadfar F, Roshandel G, Karimkhani C, Bazargan-Hejazi S, Kiadaliri AA, Ahmadieh H (2017). Disability-adjusted life-years (DALYs) for 315 diseases and injuries and healthy life expectancy (HALE) in Iran and its neighboring countries, 1990-2015: findings from global burden of disease study 2015. Arch Iran Med.

[29] Shams M, Mohebi F, Gohari K, Masinaei M, Mohajer B, Rezaei N, Sheidaei A, Khademioureh S, Yoosefi M, Hasan M (2021). The level and trend of road traffic injuries attributable mortality rate in Iran, 1990–2015: a story of successful regulations and a roadmap to design future policies. BMC Public Health.

[30] Mock C, Quansah R, Krishnan R, Arreola-Risa C, Rivara F (2004). Strengthening the prevention and care of injuries worldwide. Lancet.

[31] Khorasani-Zavareh D, Mohammadi R, Khankeh HR, Laflamme L, Bikmoradi A, Haglund BJ (2009). The requirements and challenges in preventing of road traffic injury in Iran. A qualitative study. BMC Public Health.

[32] Bakhtari Aghdam F, Sadeghi-Bazargani H, Azami-Aghdash S, Esmaeili A, Panahi H, Khazaee-Pool M, Golestani M (2020). Developing a national road traffic safety education program in Iran. BMC Public Health.

[33] Ghazizadeh-Hashemi S, Larijani B (2015). National action plan for prevention and control of non communicable diseases and the related risk factors in the Islamic Republic of Iran, 2015–2025.

[34] Salari H, Motevalian SA, Mohammad A, Esfandiari A, Sari AA (2017). Exploring measures to control road traffic injuries in Iran: key informants points of view. Iran J Public Health.

[35] Al-Thani H, El-Menyar A, Asim M, Mollazehi M, Abdelrahman H, Parchani A, Consunji R, Castle N, Ellabib M, Al-Hassani A (2019). Evolution of the Qatar trauma system: the journey from inception to verification. J Emerg Trauma Shock.

[36] Consunji R, Mekkodathil A, Abeid A, El-Menyar A, Al-Thani H, Sekayan T, Peralta R (2018). Applying the five-pillar matrix to the decade of action for road safety in Qatar: identifying gaps and priorities. Trauma Surg Acute Care Open.

[37] Global status report on road safety 2018: summary. Geneva: World Health Organization; 2018 (WHO/NMH/NVI/18.20). Licence: CC BY-NC-SA 3.0 IGO). Available at https://www.who.int/publications/i/item/WHO-NMH-NVI-18.20. Accessed 6 June 2022.

[38] Hyder AA, Allen KA, Di Pietro G, Adriazola CA, Sobel R, Larson K, Peden M (2012). Addressing the implementation gap in global road safety: exploring features of an effective response and introducing a 10-country program. Am J Public Health.

[39] GBD 2015 Eastern Mediterranean Region Neonatal, Infant, and under-5 Mortality Collaborators. Neonatal, infant, and under-5 mortality and morbidity burden in the Eastern Mediterranean region: findings from the Global Burden of Disease 2015 study. Int J Public Health 2018; 63(Suppl 1):63–77. 10.1007/s00038-017-0998-x PMC5702263 28776242

[40] Motala S, Ngandu S, Mti S, Arends F, Winnaar L, Khalema E, et al. Millennium development goals: country report 2015. Pretoria: Statistics South Africa, 2015. Available at https://repository.hsrc.ac.za/handle/20.500.11910/9580. Accessed 6 June 2022.

[41] Daemi A, Ravaghi H, Jafari M (2019). Risk factors of neonatal mortality in Iran: a systematic review. Med J Islam Repub Iran.

[42] Jenabi E, Khazaei S (2020). Child mortality rate in Iran compared with other eastern mediterranean countries based on WHO report in 2017. Iran J Public Health.

[43] Zalvand R, Tajvar M, Pourreza A, Asheghi H (2019). Determinants and causes of maternal mortality in Iran based on ICD-MM: a systematic review. Reprod Health.

[44] Marandi SA, Farrokhzad N, Moradi R, Rezaeizadeh G, Shariat M, Nayeri FS (2019). Present status of the Iranian newborns’ health, survival, and care and future challenges. Arch Iran Med.

[45] Azami-Aghdash S, Ghojazadeh M, Dehdilani N, Mohammadi M (2014). Prevalence and causes of cesarean section in Iran: systematic review and meta-analysis. Iran J Public Health.

[46] Jadoon B, Mahaini R, Gholbzouri K. Determinants of over and underuse of caesarean births in the eastern Mediterranean region: an updated review. East Mediterr Health J. 2019;25(11):837–846. 10.26719/emhj.19.033 31782521

[47] Karamouzian M, Sharifi H, Haghdoost AA (2014). Iran’s shift in family planning policies: concerns and challenges. Int J Health Policy Manag.

[48] Johnson CO, Nguyen M, Roth GA, Nichols E, Alam T, Abate D, Abd-Allah F, Abdelalim A, Abraha HN, Abu-Rmeileh NM (2019). Global, regional, and national burden of stroke, 1990–2016: a systematic analysis for the global burden of disease study 2016. Lancet Neurol.

[49] Shabestari AN, Moghaddam SS, Sharifi F, Fadayevatan R, Nabavizadeh F, Delavari A, Jamshidi HR, Naderimagham S (2015). The most prevalent causes of death, DALYs, and geriatric syndromes in Iranian elderly people between 1990 and 2010: findings from the global burden of disease 2010 study. Arch Iran Med.

[50] Sarrafzadegan N, Mohammmadifard N (2019). Cardiovascular disease in Iran in the last 40 years: prevalence, mortality, morbidity, challenges and strategies for cardiovascular prevention. Arch Iran Med.

[51] Turk-Adawi K, Sarrafzadegan N, Fadhil I, Taubert K, Sadeghi M, Wenger NK, Tan NS, Grace SL (2018). Cardiovascular disease in the eastern Mediterranean region: epidemiology and risk factor burden. Nat Rev Cardiol.

[52] Doshmangir L, Bazyar M, Rashidian A, Gordeev VS (2021). Iran health insurance system in transition: equity concerns and steps to achieve universal health coverage. Int J Equity Health.

[53] Atun R (2015). Transforming Turkey's health system—lessons for universal coverage. N Engl J Med.

[54] Younis M, Al-Hajeri M, Celik Y, Kisa A, Richard P, Parkash J (2015). Healthcare of aging population of Kuwait. Ageing Int.

[55] Goodman A (2015). The development of the Qatar healthcare system: a review of the literature. Int J Clin Med.

[56] Out-of-pocket expenditure (% of current health expenditure) https://data.worldbank.org/indicator/SH.XPD.OOPC.CH.ZS.

[57] Global Health Repository: Completeness of cause of death data https://www.who.int/data/gho/data/indicators/indicator-details/GHO/completeness-of-cause-of-death-data-(−).

[58] Roth GA, Abate D, Abate KH, Abay SM, Abbafati C, Abbasi N, Abbastabar H, Abd-Allah F, Abdela J, Abdelalim A (2018). Global, regional, and national age-sex-specific mortality for 282 causes of death in 195 countries and territories, 1980–2017: a systematic analysis for the global burden of disease study 2017. Lancet.

[59] Bhalla K, Harrison JE (2015). GBD-2010 overestimates deaths from road injuries in OECD countries: new methods perform poorly. Int J Epidemiol.

[60] Maher C, Ferreira G. Time to reconsider what global burden of disease studies really tell us about low back pain. Ann Rheum Dis. 2021;81(3):306–08. 10.1136/annrheumdis-2021-221173 PMC8862017 34583922

[61] Von der Lippe E, Devleesschauwer B, Gourley M, Haagsma J, Hilderink H, Porst M, Wengler A, Wyper G, Grant I (2020). Reflections on key methodological decisions in national burden of disease assessments. Arch Public Health.

[62] Peykari N, Hashemi H, Dinarvand R, Haji-Aghajani M, Malekzadeh R, Sadrolsadat A, Sayyari AA, Asadi-Lari M, Delavari A, Farzadfar F (2017). National action plan for non-communicable diseases prevention and control in Iran; a response to emerging epidemic. J Diabetes Metab Disord.

